# Dysregulated circular RNAs in medulloblastoma regulate proliferation and growth of tumor cells via host genes

**DOI:** 10.1002/cam4.1613

**Published:** 2018-11-06

**Authors:** Tao Lv, Yi‐Feng Miao, Ke Jin, Shuo Han, Tian‐Qi Xu, Zi‐Long Qiu, Xiao‐Hua Zhang

**Affiliations:** ^1^ Department of Neurosurgery Ren Ji Hospital Schoolof Medicine Shanghai Jiao Tong University Shanghai China; ^2^ Institute of Neuroscience State Kay Laboratory of Neuroscience CAS Center for Excellence in Brain Science and Intelligence Technology Chinese Academy of Sciences Shanghai China

**Keywords:** circular RNAs, invasion, medulloblastoma, migration, proliferation

## Abstract

Circular RNAs (circRNAs) have been demonstrated to be involved in various biological processes. Nevertheless, the function of circRNAs in medulloblastoma (MB) is still unknown. The present study aimed to investigate the expression profiles of circRNAs and related mechanisms for regulating the proliferation and growth of tumor cells in MB. The expression profiles of circRNAs were screened from four normal cerebellum and four MB samples using a HiSeq Sequencer. Bioinformatic analysis was employed to predict the interaction between circRNAs and mRNAs in MB. Subsequently, the expression levels of eight differential circRNAs [circ‐SKA3 (hsa_circ_0029696), circ‐DTL (hsa_circ_0000179), circ‐CRTAM, circ‐MAP3K5 (hsa_circ_0006856), circ‐RIMS1‐1 (hsa_circ_0132250), circ‐RIMS1‐2 (hsa_circ_0076967), circ‐FLT3‐1 (hsa_circ_0100165), and circ‐FLT3‐2 (hsa_circ_0100168)] were validated using quantitative reverse transcription−polymerase chain reaction. Moreover, circ‐SKA3 and circ‐DTL were silenced using small interfering RNAs and their host genes were overexpressed to investigate their role in the pathogenesis of MB. A total of 33 circRNAs were found to be differentially expressed in MB tissues (fold change ≥ 2.0, FDR <0.05), of which three were upregulated and 30 were downregulated; six circRNAs were experimentally validated successfully. Upregulated circ‐SKA3 and circ‐DTL promoted the proliferation migration and invasion in vitro by regulating the expression of host genes. This novel study exploited the profiling of circRNAs in MB and demonstrated that circ‐SKA3 and circ‐DTL were crucial in the tumorigenesis and development of MB and might be considered as novel and potential biomarkers for the diagnosis and new targets for the intervention of MB.

## 1 INTRODUCTION

Medulloblastoma (MB) is one of the most common malignant brain tumors, accounting for 8%‐10% of cancers in the central nervous system in children.^1^ Next‐generation sequencing technologies and genome‐wide association analysis promoted MB research to the advanced edge of cancer genomics. The recent revised fourth edition of the World Health Organization Classification of Tumors of the Central Nervous System classified MB into four distinct subgroups [Wnt, sonic hedgehog signaling (Shh), Group 3, and Group 4].^2^ Molecular signatures predict the clinical behaviors of MB more accurately than histologic criteria.^3^ Current treatments for MB are mainly based on surgical resection and external beam radiotherapy combined with chemotherapy, which account for excellent overall 5‐year survival rates (85% for average risk and 70% for high risk) in MB.^4^, ^5^, ^6^ However, striking heterogeneity of genomic alterations across patients in different subgroups or even within tumor tissues of one individual patient may lead to resistance to chemotherapies rapidly. Moreover, radiotherapy and chemotherapy are devastating to the life quality of the survivors.^7^ Developing suitable treatments with fewer side effects for the right subgroups is critical to increase survival rates and improve the life quality of survivors.

Genetic mutations, DNA methylation, histone modification, and noncoding RNAs (ncRNAs) have been indicated to be crucial in the tumorigenesis of MB.^8^, ^9^, ^10^, ^11^, ^12^ The microRNAs (miRNAs) and long noncoding RNAs (lncRNAs) are particularly important for the functional relevance of ncRNAs.^13^ Over the last few years, increasing evidence suggest that ncRNAs can be used as biomarkers for the early diagnosis and long‐term survival prediction of MB. Moreover, the expression profiles of specific ncRNAs are valuable for tumor classification, targeted therapy, and individualized intervention.^14^, ^15^, ^16^

Circular RNAs (circRNAs) are a novel class of ncRNAs, which form a closed continuous loop without 5′ caps and 3′ tails.^17^ Previously, circRNAs were considered as the by‐products of aberrant RNA splicing without clear functions. With advances in high‐throughput sequencing and bioinformatic analysis, accumulated evidence indicated that circRNAs were abundant in mammalian cells and even highly expressed compared with the linear RNA isoforms of the host genes.^18^ Recent evidence indicated that the aberrant expression profile of circRNAs occurs in colorectal cancer, bladder carcinoma, breast cancer, and glioblastoma.^19^, ^20^, ^21^, ^22^ Therefore, circRNAs are important in the tumorigenesis of various kinds of cancers. CircRNAs are found to be involved in miRNA response elements and regulated gene expression. For instance, circ_001569 acts as a powerful miR‐145 sponge/inhibitor and is vital in the cell proliferation and invasion of colorectal cancer.^23^ Another study found that the silencing of circ‐ZNF292 inhibited tube formation in human glioma cell lines.^24^

However, the expression profile and potential function of circRNAs in MB remain unknown. This study aimed to exploit the expression profiles of circRNAs in MB and address the role of critical circRNAs in the pathogenesis and development of MB. This study provided evidence that two critical differentially expressed circRNAs, circ‐SKA3 and circ‐DTL, were crucial in promoting cell proliferation, migration, and invasion in MB, possibly through a novel mechanism of regulating the expression of host genes, thereby shedding light on target therapy and potential personal intervention for MB.

## 2 RESULTS

### 2.1 Expression profiling of mRNAs and circRNAs in MB

**Figure 1:**
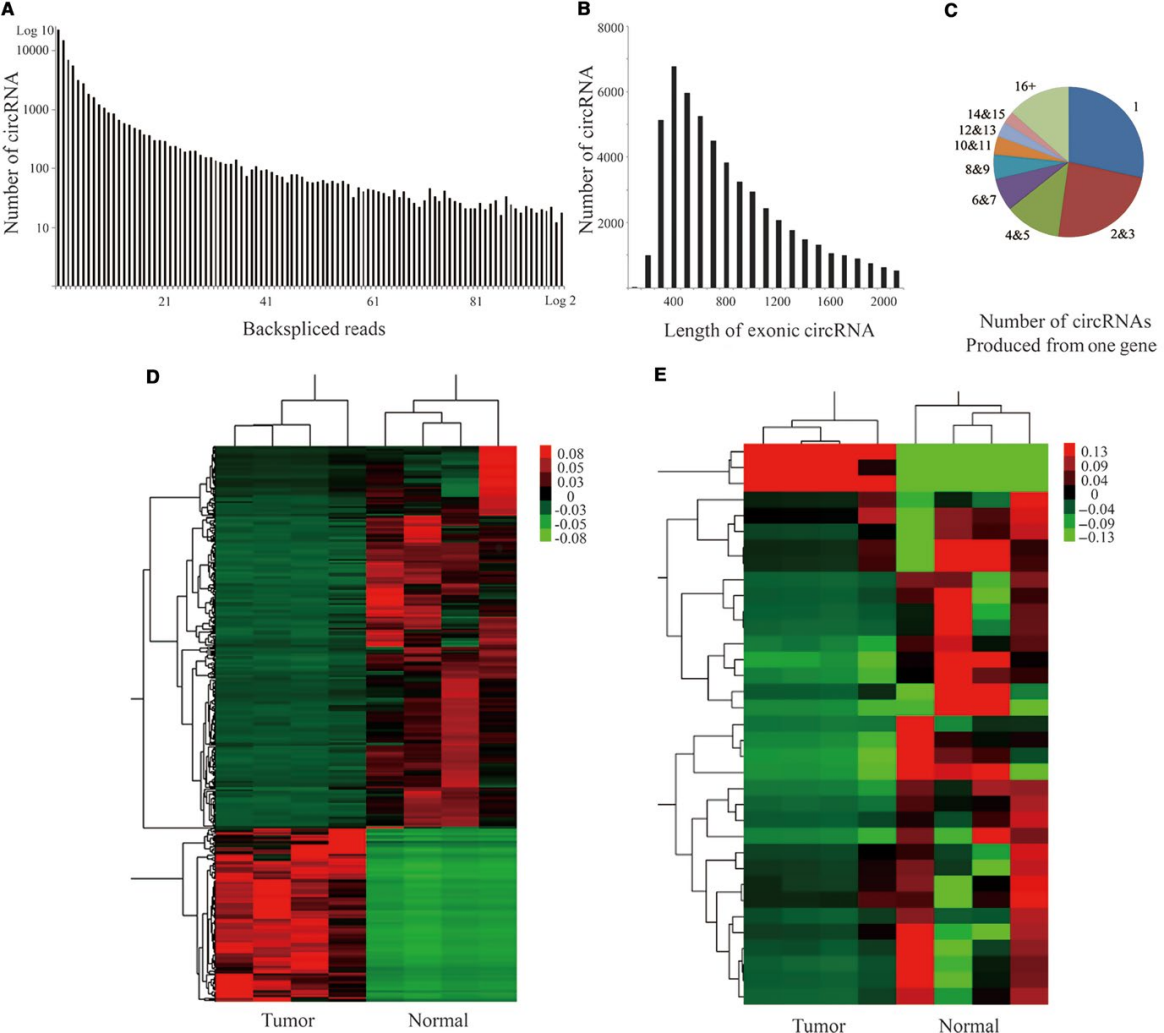
Profiling of circRNAs in human medulloblastoma (MB) and normal cerebellum tissues. A, The number of circRNAs and back‐spliced reads. B, The length distribution for exonic circRNAs. C, The number of circRNAs produced from one host gene. D, The differentially expressed mRNAs between MB and normal cerebellum tissues by hierarchical clustering analysis. E, The differentially expressed circRNAs between MB and normal cerebellum tissues by detected using hierarchical clustering analysis. Each column represents the expression profile of a sample, whereas each row represents circRNA

The next‐generation sequencing was performed on RNAs extracted from four human MB and four human normal cerebellum tissues to investigate the comprehensive expression profiles, including mRNAs and circRNAs, in MB. First, the number of circRNAs and back‐spliced reads were examined in these tissues. A total of 44 184 distinct circRNAs existed, and the back‐spliced reads of 15 638 distinct circRNAs were more than 1 (Figure 1A). The length of majority exonic circRNAs was less than 2100 nucleotides (nt), and the median length was 700 nt (Figure 1B). The results demonstrated that one host gene could produce multiple circRNAs (Figure 1C), consistent with previous findings.^17^, ^25^ The mRNAs and circRNAs were further compared in four MB and four normal cerebellum samples. As shown in Figure 1D,E, the differentially expressed mRNAs and circRNAs between MB and normal cerebellum tissues were analyzed by hierarchical clustering methods.

### 2.2 Identification of circRNA‐mRNA co‐expression network and prediction of related target genes

**Figure 2:**
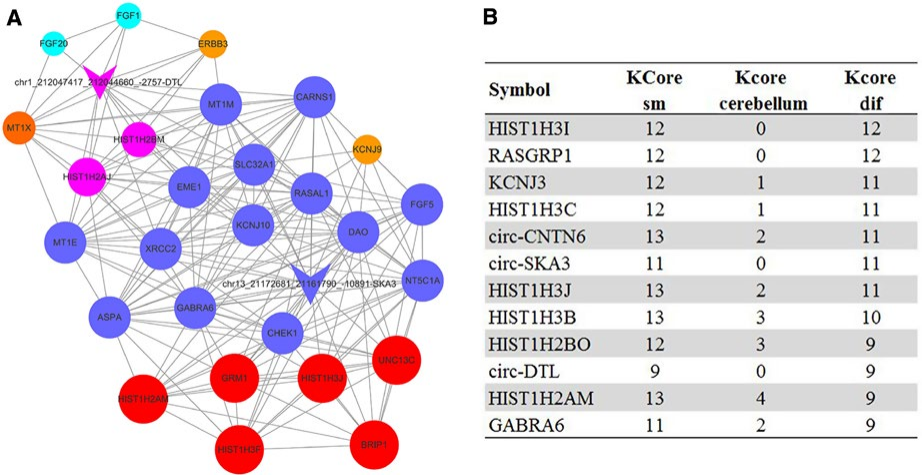
The coexpression network of gene and circRNAs. A, The coexpression network of circ‐SKA3 and circ‐DTL and related mRNAs as drawn via k‐core analysis. The circ‐SKA3 and circ‐DTL were the two circRNAs that gained the greatest k‐core difference in the network. B, The k‐core difference was calculated to identify the core gene and circRNAs in the medulloblastoma group

The coexpression networks for mRNAs and circRNAs were constructed between MB and cerebellum groups based on the expression profiling of differentially expressed circRNAs and mRNAs significantly enriched in pathway analysis (*P *<* *.05; Figure S1). The k‐core analysis was performed to discover the core mRNAs and circRNAs in those two groups, and the k‐core difference was calculated to identify the core mRNAs and circRNAs in the MB group (Figure 2B). For circRNAs, circ‐SKA3 and circ‐DTL gained the greatest k‐core difference, indicating that these two circRNAs might be important in the tumorigenesis and development of MB. The relationship of mRNA with these circRNAs was considered, and a network was constructed to conjecture the possible function of these circRNAs (Figure 2A).

### 2.3 Upregulated expression of circ‐SKA3 and circ‐DTL in MB

**Figure 3:**
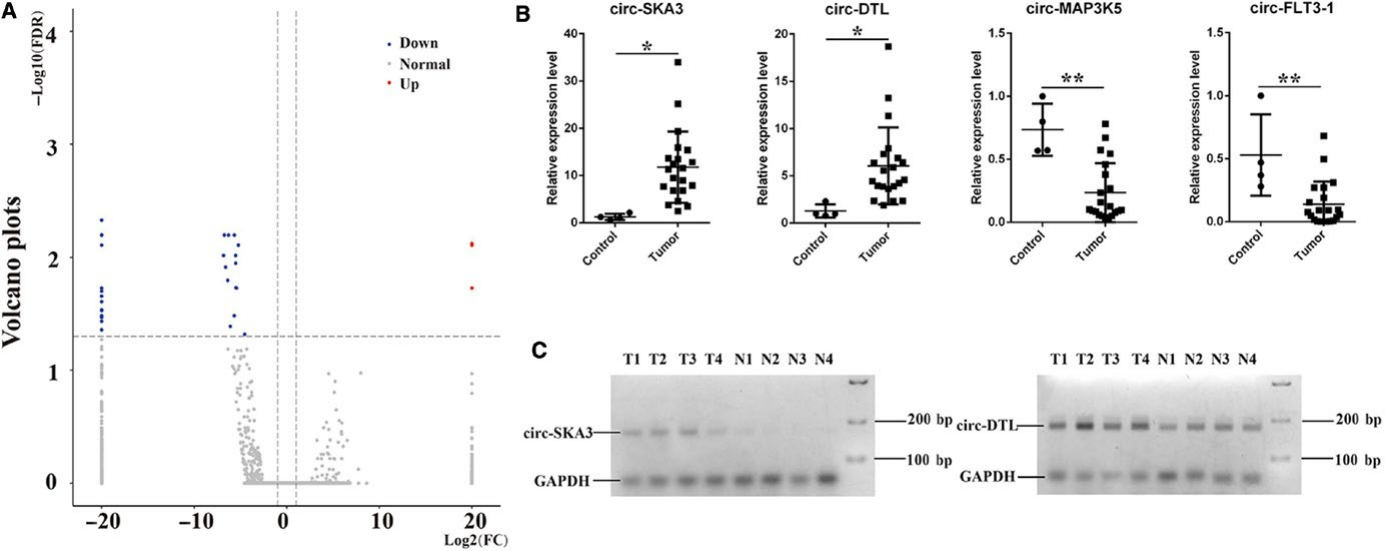
Differentially expressed circRNAs in medulloblastoma (MB). A, Volcano plot shows differentially expressed circRNAs with statistical significance. The vertical dotted lines represent 2.0‐fold change up and down. The horizontal dotted line corresponds to an FDR value of 0.05. The red points in the plot represent upregulated circRNAs, the blue points represent the downregulated circRNAs, and the gray points represent the normal circRNAs. B, The relative expression of circRNAs in MB and normal cerebellum tissues detected using qRT‐PCR. C, RT‐PCR gels show that circ‐SKA3 and circ‐DTL are enriched in MB. *P <* *.05, **P <* *.01

A total of 33 differentially expressed circRNAs were identified using the volcano plot (fold change ≥2.0 and FDR <0.05) in MB tissues compared with normal cerebellum tissues, including three upregulated and 30 downregulated circRNAs (Figure 3A, Table 1). Gene Ontology (GO) analysis revealed that the differentially expressed circRNAs participated in several critical biological processes and signaling pathways. The results are shown in Figure S2. Further, the expression levels of eight differential circRNAs (circ‐DTL, circ‐SKA3, circ‐CRTAM, circ‐MAP3K5, circ‐RIMS1‐1, circ‐RIMS1‐2, circ‐FLT3‐1, circ‐FLT3‐2) were validated by quantitative reverse transcription polymerase chain reaction (RT‐PCR) in 21 MB and four normal cerebellum samples. The expression levels of circ‐SKA3 and circ‐DTL were found to be significantly upregulated in patients with MB, whereas circ‐CRTAM, circ‐MAP3K5, circ‐RIMS1‐1, and circ‐FLT3‐1 were significantly downregulated, consistent with the results of RNA sequencing (Figures 3B and S3). RT‐PCR gels also showed that circ‐SKA3 and circ‐DTL were enriched in MB (Figure 3C).

**Table 1:** CircRNAs dysregulated in medulloblastoma

CircRNAs	ACC‐ID (Chrom_Start_End_Length)	Regulation in MB	*P*‐value	Log2 fold change	FDR	CircRNAs type	Host gene
Circ‐ SKA3	chr13_21172681_21161790_‐10891	Up	3.65E‐06	20	7.57E‐03	Exonic	SKA3
Circ‐ DTL	chr1_212047417_212044660_‐2757	Up	4.63E‐06	20	7.82E‐03	Exonic	DTL
Circ‐ CASC15	chr6_22063240_22056546_‐6694	Up	1.99E‐05	20	1.88E‐02	Intronic	CASC15
Circ‐ UNC13C	chr15_54338489_54293895_‐44594	Down	2.26E‐07	−20	4.69E‐03	Exonic	UNC13C
Circ‐ BRWD3	chrx_80736088_80733456_‐2632	Down	7.33E‐07	−20	6.36E‐03	Exonic	BRWD3
Circ‐ CNTN6	chr3_1297991_1295601_‐2390	Down	1.14E‐06	−6.75	6.36E‐03	Exonic	CNTN6
Circ‐ CRTAM	chr11_122868099_122867409_‐690	Down	1.27E‐06	−20	6.36E‐03	Exonic	CRTAM
Circ‐ MCU	chr10_72715902_72708283_‐7619	Down	1.67E‐06	−20	6.36E‐03	Intronic	MCU
Circ‐ RIMS1‐1	chr6_72333835_72307258_‐26577	Down	2.15E‐06	−5.66	6.36E‐03	Exonic	RIMS1
Circ‐ FLT3‐1	chr13_28050222_28033887_‐16335	Down	2.49E‐06	−20	6.36E‐03	Exonic	FLT3
Circ‐ DGKH	chr13_42190525_42168440_‐22085	Down	2.61E‐06	−20	6.36E‐03	Exonic	DGKH
Circ‐ FLT3‐2	chr13_28050222_28037185_‐13037	Down	4.57E‐06	−20	7.82E‐03	Exonic	FLT3
Circ‐ SPHKAP	chr2_228020156_228016406_‐3750	Down	4.9E‐06	−5.23	7.82E‐03	Exonic	SPHKAP
Circ‐ GRM1	chr6_146399699_146398769_‐930	Down	6.85E‐06	−6.83	9.65E‐03	Exonic	GRM1
Circ‐ GABRB2	chr5_161336769_161330883_‐5886	Down	6.98E‐06	−5.53	9.65E‐03	Exonic	GABRB2
circ‐ SYNE1	chr6_152628554_152628265_‐289	Down	8.7E‐06	−5.51	1.12E‐02	Exonic	SYNE1
Circ‐ UNC13C	chr15_54250444_54235030_‐15414	Down	1.0E‐05	−6.60	1.12E‐02	Exonic	UNC13C
Circ‐ RIMS1‐2	chr6_72251368_72250330_‐1038	Down	1.39E‐05	−6.38	1.60E‐02	Exonic	RIMS1
Circ‐ ICA1	chr7_8236005_8218305_‐17700	Down	1.84E‐05	−5.43	1.88E‐02	Exonic	ICA1
Circ‐ GRIK2	chr6_101818483_101799648_‐18835	Down	1.94E‐05	−20	1.88E‐02	Exonic	GRIK2
Circ‐ATP8A2	chr13_25839624_25828118_‐11506	Down	2.22E‐05	−20	2.00E‐02	Exonic	ATP8A2
Circ‐ EPHX2	chr8_27536855_27525362_‐11493	Down	2.56E‐05	−20	2.21E‐02	Exonic	EPHX2
Circ‐ WAC	chr10_28596041_28583399_‐12642	Down	2.99E‐05	−20	2.47E‐02	Exonic	WAC
Circ‐ TENM1	chrx_124896241_124894296_‐1945	Down	3.67E‐05	−20	2.92E‐02	Exonic	TENM1
Circ‐ SNORD109A	chr15_25042515_25039711_‐2804	Down	3.85E‐05	−20	2.96E‐02	UnKnown	SNORD109A
Circ‐ UNC13C	chr15_54265496_54235030_‐30466	Down	3.99E‐05	−20	2.96E‐02	Exonic	UNC13C
Circ‐ GRIK2	chr6_101928632_101799648_‐128984	Down	4.75E‐05	−20	3.30E‐02	Exonic	GRIK2
Circ‐ MAP3K5	chr6_136698682_136694140_‐4542	Down	4.77E‐05	−5.67	3.30E‐02	Exonic	MAP3K5
Circ‐ CAMKK2	chr12_121248734_121244573_‐4161	Down	5.08E‐05	−20	3.40E‐02	Exonic	CAMKK2
Circ‐ SVEP1	chr9_110404552_110400854_‐3698	Down	5.69E‐05	−20	3.68E‐02	Exonic	SVEP1
Circ‐ CADPS2	chr7_122474517_122407540_‐66977	Down	6.51E‐05	−6.10	4.10E‐02	Exonic	CADPS2
Circ‐ CAMK4‐1	chr5_111394782_111374850_‐19932	Down	7.2E‐05	−20	4.40E‐02	Exonic	CAMK4
Circ‐ CAMK4‐2	chr5_111394782_111344024_‐50758	Down	8.11E‐05	−4.54	4.81E‐02	Exonic	CAMK4

### 2.4 Effect of circ‐SKA3 and circ‐DTL in regulating the expression of SKA3 and DTL

**Figure 4:**
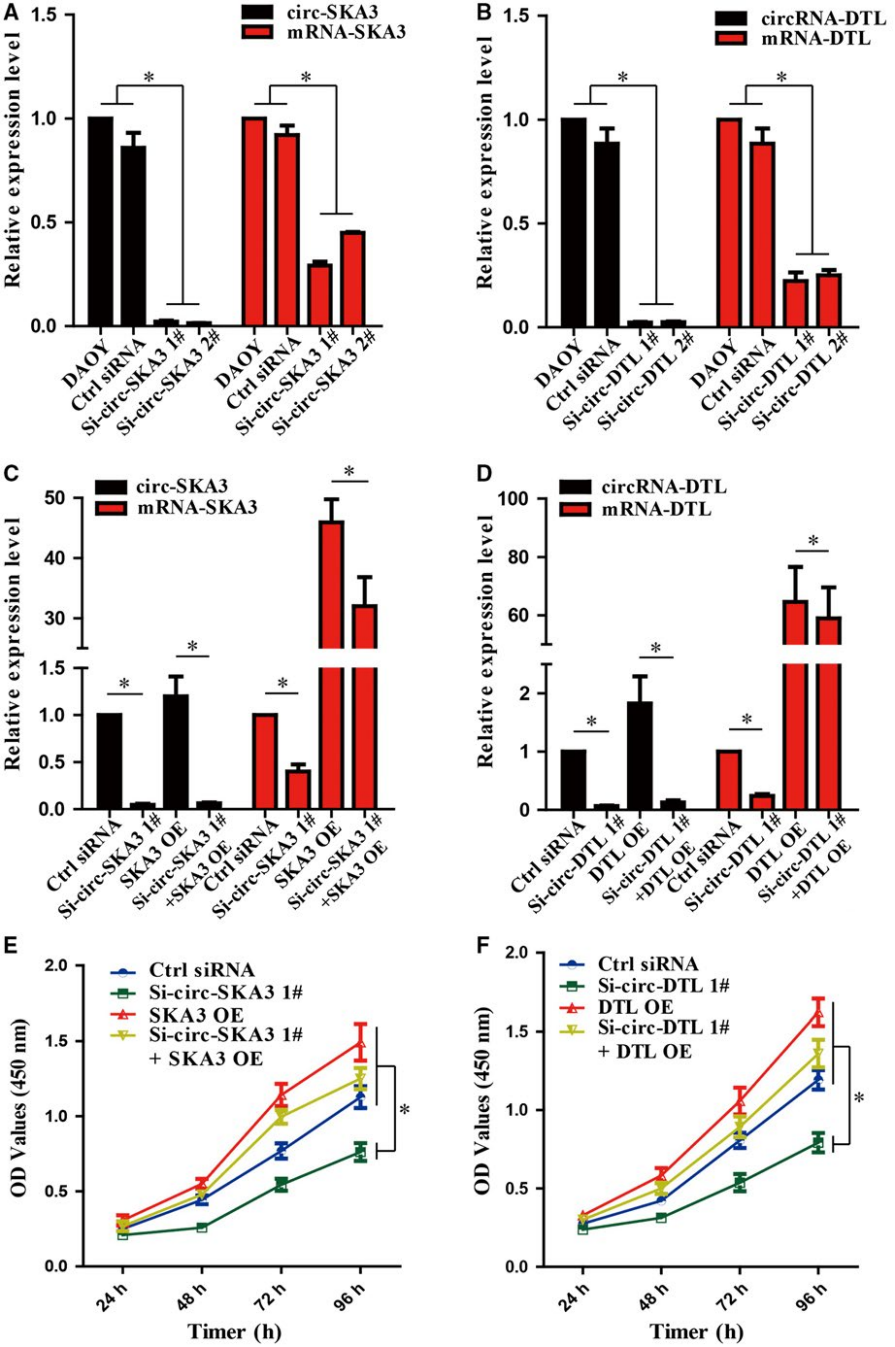
Effect of circ‐SKA3 and circ‐DTL on the proliferation of DAOY cell line in vitro. A, The effect of Si‐circ‐SKA3 plasmids on the expression of circ‐SKA3. B, The effect of Si‐circ‐DTL plasmids on the expression of circ‐DTL. C, The effect of circ‐SKA3 silencing and overexpression of linear transcript of SKA3 on the expression of circ‐SKA3 and linear transcript of SKA3. D, The effect of circ‐DTL silencing and overexpression of linear transcript of DTL on the expression of circ‐SKA3 and linear transcript of DTL. E, The effect of circ‐SKA3 silencing and overexpression of mRNA of SKA3 on the proliferation potential of the medulloblastoma (MB) DAOY cell line. F, The effect of circ‐DTL silencing and overexpression of mRNA of DTL on the proliferation potential of the MB DAOY cell line. The data are expressed as means ± standard deviation; *P <* *.05

Small interfering RNA (siRNA) constructs were designed against circ‐SKA3 and circ‐DTL to silence the expression of circ‐SKA3 and circ‐DTL, so as to investigate the effect of circ‐SKA3 and circ‐DTL in the pathogenesis and development of MB. After transfection of Si‐circ‐SKA3 and Si‐circ‐DTL along with control siRNA into the DAOY cell lines, siRNAs against circRNAs was found to significantly decrease the expression of circ‐SKA3 and circ‐DTL, as well as their host genes, SKA3 and DTL (Figure 4A,B). Additionally, on overexpressing the mRNAs of SKA3 and DTL, the expression of circ‐SKA3 and circ‐DTL did not change significantly (Figure 4C,D). Therefore, this study suggested that the expression of circ‐SKA3 and circ‐DTL directly influenced the expression of host genes, SKA3 and DTL. Given the function of circRNAs so far, the major role of circRNAs was to absorb miRNAs, rather than directly affecting the expression of their host genes. Therefore, this study suggested that circRNAs might exhibit physiological functions by controlling the expression of their host genes.

### 2.5 Circ‐SKA3 and circ‐DTL regulated proliferation migration and invasion of tumor cells by controlling the expression of SKA3 and DTL

Si‐circ‐SKA3 and Si‐circ‐DTL were transfected along with control siRNA, with or without their host genes, into the DAOY cell lines and the growth of tumor cells under various conditions was measured to examine the effect of circ‐SKA3 and circ‐DTL in the growth of tumor cell lines. Downregulating the expression of circ‐SKA3 and circ‐DTL with siRNA plasmids significantly suppressed the proliferation of DAOY cell line (Figure 4E,F). However, the proliferation ability of DAOY cell line was recovered when SKA3 and DTL were coexpressed, suggesting that inhibiting the host gene is the mechanism by which circRNAs regulate the proliferation of tumor cells (Figure 4E,F).

**Figure 5:**
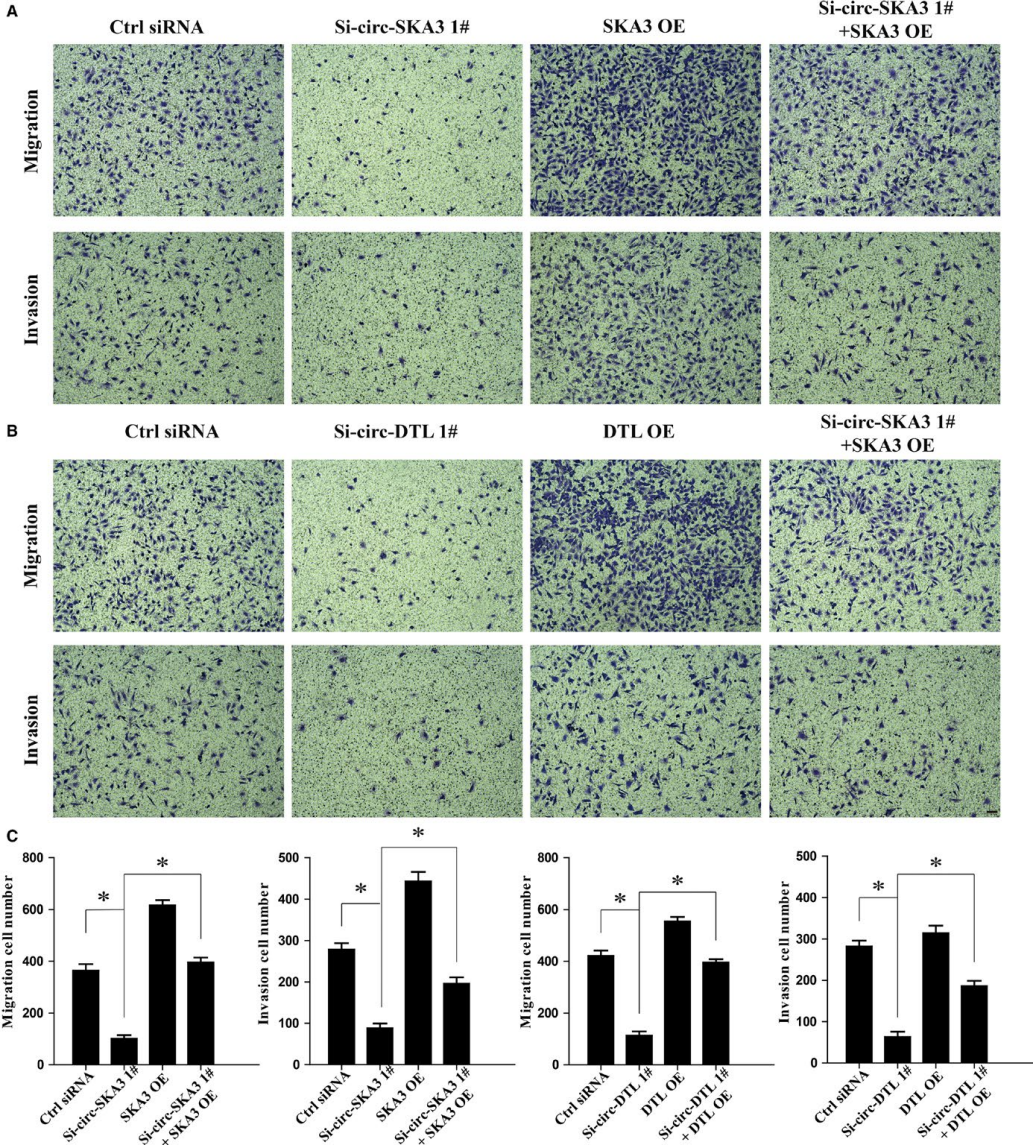
Effect of circ‐SKA3 and circ‐DTL on the migration and invasion capacity of DAOY cell line in vitro. A, Circ‐SKA3 silencing reduced the migration and invasion capacity of DAOY cells in Transwell migration assays, while overexpression of mRNA of SKA3 recovered the migration and invasion ability. B, Circ‐DTL silencing and overexpression of mRNA of DTL showed the same trend. C, The quantification number of migration and invasion cells with different expression levels of circRNAs and mRNAs. Scale bars represent 50 μm. Magnification 100×; data expressed as means ± standard deviation; *P <* *.05

The migration and invasion of DAOY cells were further investigated using the Transwell assay with various manipulations. The knockdown of circ‐SKA3 or circ‐DTL significantly inhibited the migration and invasion capacity of DAOY cells compared with controls (Figure 5). Interestingly, the effect of Si‐circ‐SKA3 or Si‐circ‐DTL on the migration and invasion of tumor cells was fully rescued by the coexpression of SKA3 or DTL (Figure 5). These evidence strongly suggested that circ‐SKA3 and circ‐DTL promoted the motility, migration, and invasion capacity of DAOY cells, importantly, through the regulation of their host genes, spindle‐kinetochore associated (SKA) and DTL. Taken together, the findings of the present study strongly suggested that upregulated circRNAs in MB, such as circ‐SKA3 or circ‐DTL, play oncogenic roles in promoting tumorigenesis. The novel mechanisms depending on host genes might lead to the development of potential therapeutic interventions for MB.

## 3 DISCUSSION

Emerging evidence indicates that ncRNAs, including miRNAs, lncRNAs, and circRNAs, are transcribed in mammals pervasively. At present, miRNAs and lncRNAs are the most studied ncRNAs, and their dysregulated expression serves as a potential biomarker and intervention target for cancer.^26^, ^27^, ^28^ The existence of circRNAs was indicated decades ago. Nevertheless, it was considered to be rare and meaningless.^29^ CircRNAs are covalently closed, comprise exonic sequences typically, and splice at specific splice sites.^30^ The biogenesis of circRNAs is cotranscriptional and posttranscriptional. Previous studies found that flanking long introns, complementary repeats, and RNA‐binding proteins, such as adenosine to inosine acting on RNA enzyme, quaking I 5, and muscleblind, are positively associated with the biogenesis of circRNAs.^31^, ^32^ With the rapid advances in high‐throughput RNA sequencing and bioinformatic analysis, circRNAs have attracted immense attention owing to their involvement in the development of various diseases. Increasing studies have revealed that circRNAs are involved in transcriptional and posttranscriptional regulation, and their cellular localization is determinant to physiological function and functional mechanism.^33^ Moreover, circRNAs are associated with the initiation and promotion of different kinds of cancers.

The present study reported the dysregulation of circRNAs in tumor samples was in patients with MB and explored the potential role of circRNAs in the pathogenesis and development of MB. Most circRNAs were downregulated in MB tissues, similar to breast cancer, hepatocellular carcinoma, laryngeal cancer, colorectal cancer, gastric cancer, and prostate adenocarcinoma reported by several previous studies.^25^, ^34^, ^35^ In this study, a total of 36 differentially expressed circRNAs were observed in tumor tissues from patients with MB, including 33 downregulated circRNAs and three upregulated circRNAs. The results of this study demonstrated that the differentially expressed circRNAs participated in several biological processes and signaling pathways. The downregulated circRNAs were associated with synaptic transmission, detection of tumor cells, and activation of mitogen‐activated protein kinase (MAPK) via GO analysis. A previous study showed that the MAPK pathway activation was crucial in drug resistance and tumor evolution of patients with Shh pathway‐dependent MB.^36^ The upregulated circRNAs were associated with G2 DNA damage checkpoint, chromosome segregation, protein monoubiquitination, protein binding, and ubiquitin‐protein transfer. CircRNAs interacted with RNA‐binding proteins to form large RNA‐protein complexes, which might regulate the pool of RNA‐binding proteins.^37^ Previous studies have shown that SKA3 and DTL are important in the development and progression of cancers.^38^, ^39^, ^40^, ^41^ However, no study has reported the function of circular transcriptomes produced from the SKA3 and DTL genes. According to the sequencing and RT‐PCR results, this study hypothesized that the upregulated circ‐SKA3 and circ‐DTL were involved in the tumorigenesis and development of MB.

SKA3, also termed C13Orf 3, is a novel member of the SKA complex and required for timely progression from metaphase to anaphase. SKA3 can map the interaction domains among the SKA components and localize to the spindle and kinetochore throughout mitosis.^42^ SKA3 is essential in cell division, and the depletion of SKA3 delays anaphase transition.^43^, ^44^ SKA3 is found to be somatically mutated in breast cancer and is important for cell growth.^39^ Overexpression of SKA3 is also confirmed in adenocarcinoma tissues, and knockdown of SKA3 in colorectal cancer cells significantly reduced cell growth rates and increased apoptosis.^38^ DTL, also known as CDT2/RAMP/DCAF2/L2DTL, is located on chromosome 1q32, which is amplified in various human solid cancers.^45^ The protein encoded by DTL gene contains six highly conserved WD40‐repeat domains and a consensus nuclear‐localization signal. It can mediate the polyubiquitination and subsequent degradation of CDT1, p12, p21, SET8, and Gcn5 and interact with Cullin‐RING ubiquitin ligases 4 (CRL4) complex.^40^ Overexpression of DTL has been demonstrated to enhance the proliferative and invasive capability and metastatic potential.^46^, ^47^, ^48^ Zhaoyong Li and colleagues speculated the presence of a positive feedback in the process of gene's transcription between exon‐intron circRNAs (EIciRNAs) and the parental genes. They found that the knockdown of circ‐EIF3J and circ‐PAIP2 with siRNAs resulted in a decrease in the circRNAs and mRNA levels of the parental genes, indicating that EIciRNAs localized in the nucleus would regulate the transcription of their parental genes through RNA‐RNA interaction between U1 snRNA and EIciRNAs.^49^ Based on this evidence, this study hypothesized that upregulated circ‐SKA3 and circ‐DTL would regulate the expression of host genes and be involved in the tumorigenesis and development of MB.

This study supposed that upregulated circ‐SKA3 and circ‐DTL might promote tumor cell proliferation and survival, migration capacity, and invasion ability in MB cells. To verify this hypothesis, Si‐circ‐SKA3 and Si‐circ‐DTL plasmids were constructed to silence the expression of circ‐SKA3 and circ‐DTL. Then, the mRNA of SKA3 and DTL was overexpressed. This study demonstrated that siRNA plasmids decreased the expression of circ‐SKA3 and circ‐DTL and mRNA of SKA3 and DTL. Moreover, the downregulation of expression of circ‐SKA3 and circ‐DTL suppressed cell proliferation, migration, and invasion in the DAOY cell lines. Furthermore, the proliferation, migration, and invasion of DAOY cells were recovered by rescue with the overexpressed plasmid of mRNAs of host genes.

The results of this study indicated that circ‐SKA3 and circ‐DTL were essential in tumor cell proliferation, migration, and invasion through regulating the expression of the host genes. In conclusion, this novel study suggested that circ‐SKA3 and circ‐DTL had a pivotal oncogenic effect in the tumorigenesis and development of MB. Moreover, these two upregulated circRNAs were important novel molecular markers for diagnosis and targets for intervention in patients with MB.

## 4 METHODS

### 4.1 Whole transcriptome sequencing and data analysis

The total RNA was extracted from frozen tissues (stored at −80°C) using the TRIzol reagent (Invitrogen, CA, USA) according to manufacturer's protocol. The RIN >8.0 was used to construct ribosomal RNA depletion library [VAHTSTM Total RNA‐seq (H/M/R)] according to the manufacturer's instructions. Whole transcriptome sequencing data sequenced using the HiSeq Sequencer was filtered [removing the adaptor sequences, reads with >5% ambiguous bases (noted as N) and low‐quality reads containing more than 20% of bases with a quality of <20] and mapped to the human genome (GRCH38.p2 NCBI) using HISAT2. HTSeq was used to calculate the gene count of mRNAs and circRNAs.

### 4.2 CircRNA prediction and functional analysis

CircRNA was predicted based on the sequencing data using ACFS pipeline.^48^ Unmapped reads were collected using BWA‐mem (BWA mem ‐T 1 ‐K 16 ‐T 20) for circRNA identification: head‐to‐tail junction was identified, and highest splicing strength score was calculated using MaxEntScan33 with a filtering criterion greater than or equal to 10. Based on the realignment of the unmapped reads on the circRNA candidates, reads that mapped to the circRNA back‐splicing junction (with an overhang of at least 6 nt) were counted for each candidate. The GO analysis with GO database (downloaded from NCBI, UniProt, and AmiGO) was applied to explore the functional roles of dysregulated circRNAs in terms of biological processes, cellular components, and molecular functions. Significant *P* value was defined using the Fisher's exact test, and FDR was calculated using the BH test.

### 4.3 Coexpression analysis

This study presented the gene coexpression networks to find the relationship between genes and circRNAs1. The gene coexpression networks were built according to the normalized expression values of genes selected from genes in significant GO terms and pathway terms and differentially expressed circRNAs. For each pair of genes, the Pearson correlation analysis was performed, and significant correlation pairs (FDR <0.05) were chosen to construct the network. Moreover, the k‐cores in graph theory were introduced as a method of simplifying the graph topology analysis to study some properties of the networks, which could represent the core status of the circRNA among genes and circRNAs.

### 4.4 Cell culture

Human MB DAOY cell lines were purchased from the American Type Culture Collection (VA, USA) and cultured in Dulbecco's modified Eagle's medium (DMEM) supplemented with 10% fetal bovine serum (FBS), 1% penicillin‐streptomycin, and 1 mmol/L glutamine (Invitrogen). The cells were cultured in a humidified incubator at 37°C with 5% CO_2_/5% O_2_/90% N_2_ atmosphere.

### 4.5 Cell transfection with siRNAs and plasmids

All the siRNAs were purchased from GenePharma (Shanghai, China). The mRNAs of circ‐SKA3 and circ‐DTL were synthesized and cloned into pcDNA3.1. The cells were transfected with siRNA plasmids and pcDNA3.1 using the Lipofectamine 3000 Transfection Reagent (Invitrogen). The siRNAs and primers used are listed in Table S1.

### 4.6 qRT‐PCR

After transfection with siRNA and plasmids, the total RNA of transfected cells was extracted using the TRIzol reagent according to manufacturer's protocol. Before testing the expression level of circRNAs, RNase R digestion reaction was performed on the basis of Danan M's procedures. The expression levels of circRNAs and mRNAs of host genes were analyzed using the One Step SYBR Premix Ex Taq II (TaKaRa, China). The custom‐designed opposite‐directed primers for specifically detecting circRNAs and primers for mRNAs are listed in Tables S2 and S3. All the primers for circRNAs and linear transcripts were purchased from HuaGene Biotech (Shanghai, China) and listed in Table. Glyceraldehyde 3‐phosphate dehydrogenase was used as an internal control. The relative expression of circRNAs and linear transcripts of the corresponding host genes was calculated using the 2^−ΔΔCt^ method. All data were expressed as mean ± standard deviation (SD) of three independent experiments.

### 4.7 Cell counting kit 8 cell viability assay

The cell viability was assessed using the cell counting kit 8 (CCK‐8) assay kit (Beyotime Institute of Biotechnology, Shanghai, China) according to the manufacturer's protocol. Each well of a 96‐well plate was seeded with 100 μL of transfected cell suspension (~2000 cells) harvested in the logarithmic phase, and the cell viability was detected every 24 hours. Before detection, 10 μL of CCK‐8 solution was added to each well and incubated at 37°C for 2 hours. The absorbance at 450 nm was then measured using a spectrophotometric reader.

### 4.8 Cell migration and invasion assays

DAOY cells were transfected with 100 nm siRNA plasmids or overexpressed with linear transcripts of the corresponding host gene plasmids for 48 hours before being harvested. To test migration, 2 × 104 transfected cells were plated in the upper chamber of the Transwell assay inserts (Millipore, MA, USA) containing 1 mL of serum‐free DMEM with a membrane (8‐mm pores), while the lower chambers were filled with DMEM supplemented with 10% FBS. In the invasion assay, 2 × 10^4^ transfected cells were plated in the upper chamber containing 1 mL serum‐free DMEM and a Matrigel‐coated membrane (BD Biosciences). The migration ability was determined after 24 hours, and the invasion function was determined after 48 hours. After fixing with methanol, the cells on the membrane were stained with crystal violet and photographed using a digital microscope. The cell numbers were calculated in three random fields for each chamber via ImageJ software.

### 4.9 Statistical analysis

All values were presented as mean ± standard deviation. The relative expression of circRNAs between normal cerebellum and MB samples verified via qRT‐PCR was analyzed using the independent‐sample *t* test. The statistical analyses between groups were performed by one‐way analysis of variance followed by the Bonferroni test using the SPSS software package (version 20.0, SPSS Inc.) and GraphPad Prism 5.0 statistical software. A *P* value <.05 was considered statistically significant.

## ETHICS STATEMENT AND SAMPLES COLLECTION

The present study was approved and supervised by The Ethics Committee of Renji Hospital, School of Medicine of Shanghai Jiao Tong University (2017058). Four human medulloblastoma tissues and four normal cerebellum tissues were obtained from the patients who underwent operation at Renji Hospital (Shanghai, China). We obtained written informed consents from patients for research purposes. All medulloblastoma patients was not treated with chemotherapy or radiotherapy before surgery. Two experienced pathologists confirmed the pathologic diagnosis independently.

## CONFLICT OF INTEREST

Authors declare that they do not have conflict of interests for this work.
