# Supplementary figures and images for: Dysregulated circular RNAs in medulloblastoma regulate proliferation and growth of tumor cells via host genes
Source: Cancer Med. 2018 Nov 6;7(12):6147–57. doi: 10.1002/cam4.1613 (PMC6308054; doi:10.1002/cam4.1613)

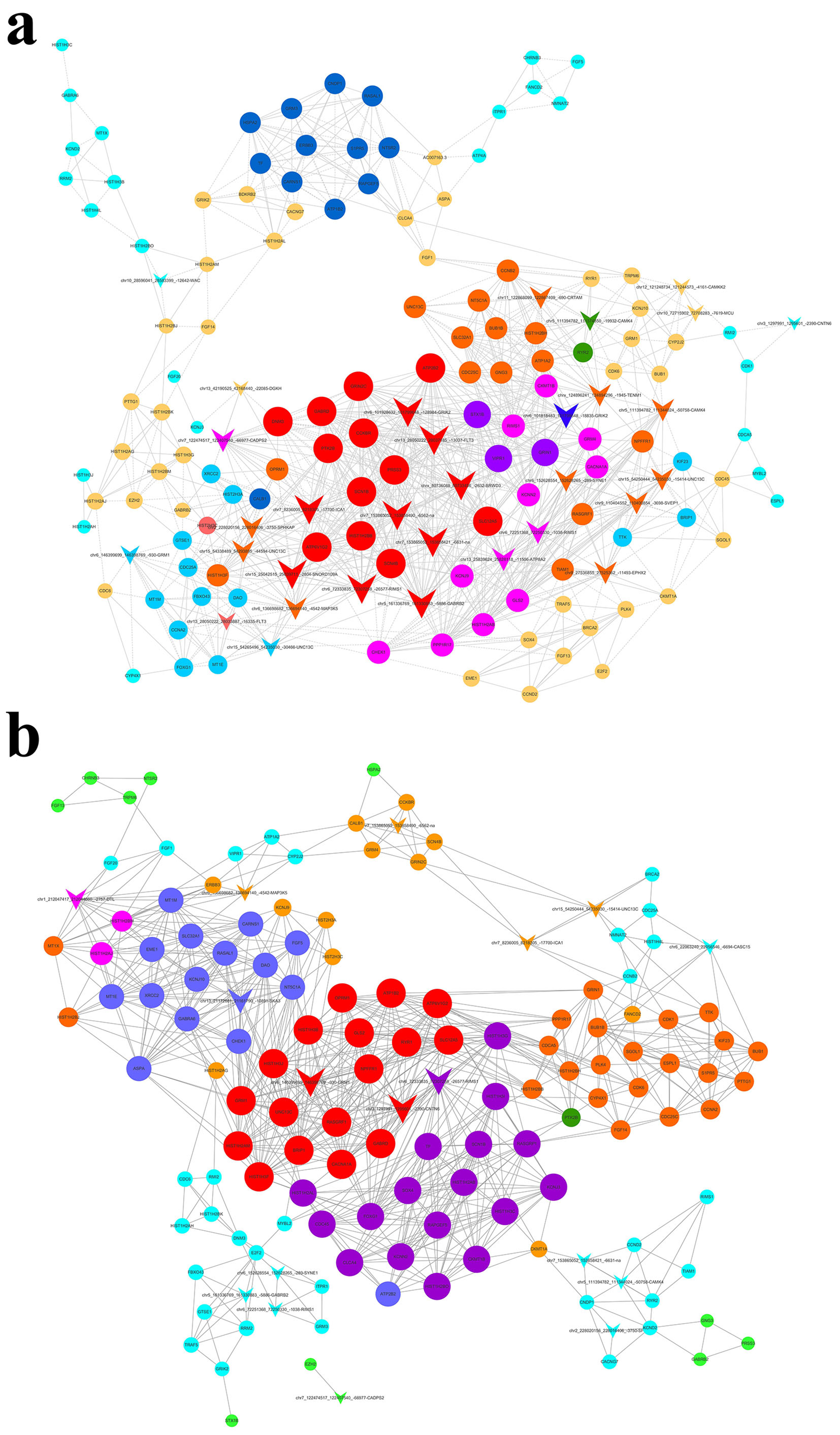

Supplement: Supplementary file 1 [file CAM4-7-6147-s001.tif]

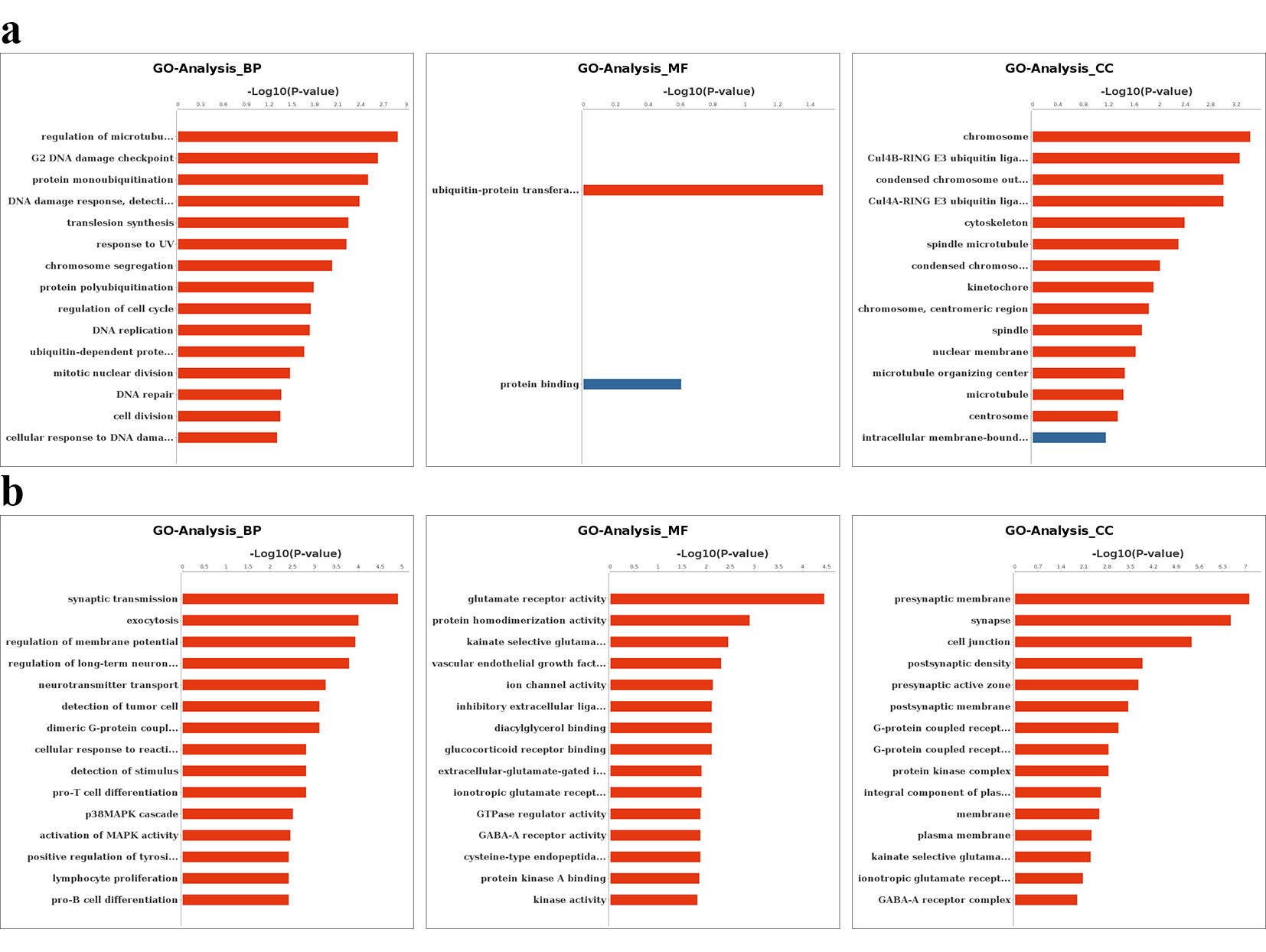

Supplement: Supplementary file 2 [file CAM4-7-6147-s002.tif]

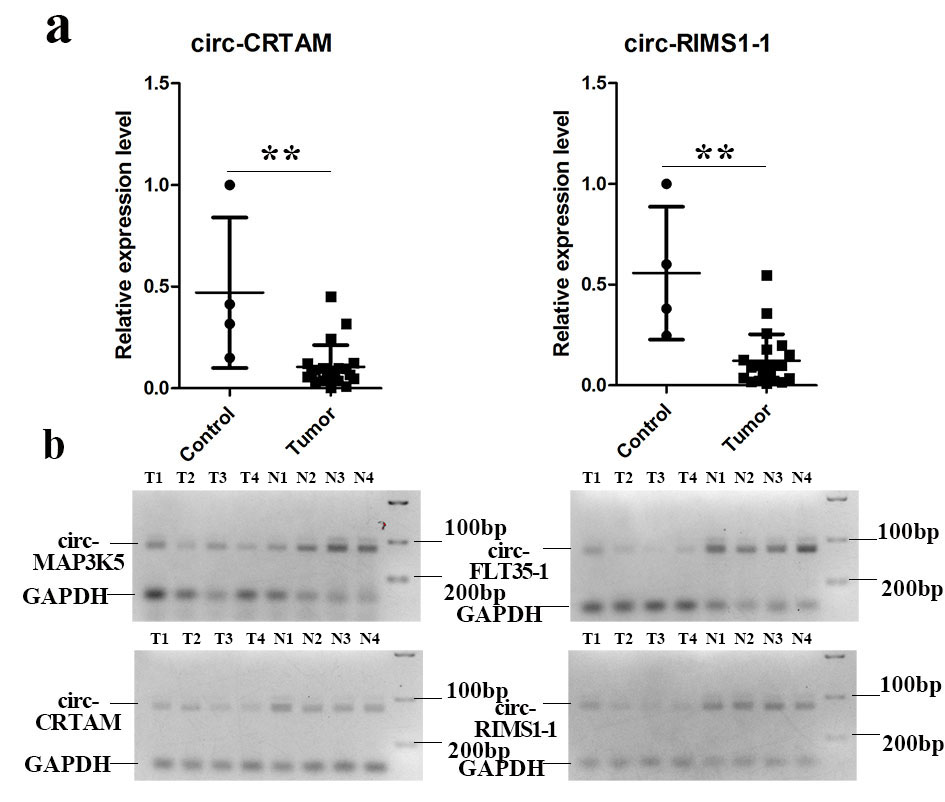

Supplement: Supplementary file 3 [file CAM4-7-6147-s003.tif]
