# Supplementary material for: Dysregulated circular RNAs in medulloblastoma regulate proliferation and growth of tumor cells via host genes
Source: Cancer Med. 2018 Nov 6;7(12):6147–57. doi: 10.1002/cam4.1613 (PMC6308054; doi:10.1002/cam4.1613)
Supplement: Supplementary file 4 [file CAM4-7-6147-s004.docx]

**Supplementary Table 1. SiRNAs for circRNAs**

| circRNAs | siRNAs (5'-3') |
| --- | --- |
| circRNA-SKA3 | Si-circRNA-SKA3-1: GTGACTTTGAAGATTATCC  Si-circRNA-SKA3-2: GTCACTTTTTTCCAACTGC |
| circRNA-DTL | Si-circRNA-DTL-1: GTTTGAGAAAGCTCCCAAT  Si-circRNA-DTL-2: AGCTCCCAATATGGAACAT |

**Supplementary Table 2. Real-time qRT-PCR primer list for circRNAs**

| circRNAs | Primer sequences (5'-3') | Annealing temperature/˚C |
| --- | --- | --- |
| GAPDH(Human) | F: AGGGCTGCTTTTAACTCTGGT  R: CCCCACTTGATTTTGGAGGGA | 60 ˚C |
| circRNA-SKA3 | F: GCCACTCCCAGCCCCATC  R: TGTTGCCTTTATGAAATCAATGCC |  |
| circRNA-DTL | F: CTAGCAGTTGCCAATGAAGAAG  R: ATTGATGACCTTTGCATGTTCC |  |
| circRNA-MAP3K5 | F: TTTGGACTCTAATTTCACGGACAC  R: AGCAGTAGACTTTGTTATGTGGAG |  |
| circRNA-CRTAM | F: CACACTAGAAAGTTACAGATCAAGG  R: ATGAAGAGTATGAAAATGAGGAAGG |  |
| circRNA-FLT3-1 | F: TGGAAAAGTGATGAACGCAACAG  R: CATTTCTGGCACAGCACCTTATG |  |
| circRNA-FLT3-2 | F: GCCCAATTTACCAAAATGTTCACG  R: CATTTCTGGCACAGCACCTTATG |  |
| circRNA-RIMS1-1 | F: ACACATGATGAGTCTTCACTACC  R: GTTGCTTGCAGAACATTTACAATC |  |
| circRNA-RIMS1-2 | F: CAGGTGGAAAGAAACGGAGATC  R: ACCCTGTTGTCTGCTTAAATCG |  |
| F: forward; R: reverse | | |

**Supplementary Table 3. Real-time qRT-PCR primer list for mRNAs**

| mRNAs | Primer sequences (5'-3') | Annealing temperature/˚C |
| --- | --- | --- |
| mRNA-SKA3 | F: ATTCAGTACACGAGCAAGAAGC  R: GCAACAGGAGGATCAGACAGA | 60 ˚C |
| mRNA-DTL | F: TAGCAGTTGCCAATGAAGAAGG  R: GCATTCCAGTGAGCCATCCA |  |
| F: forward; R: reverse | | |
